# Sustainable Use of Apple Pomace (AP) in Different Industrial Sectors

**DOI:** 10.3390/ma15051788

**Published:** 2022-02-27

**Authors:** Ewelina Gołębiewska, Monika Kalinowska, Güray Yildiz

**Affiliations:** 1 Department of Chemistry, Biology and Biotechnology, Faculty of Civil Engineering and Environmental Science, Institute of Civil Engineering and Energetics, Bialystok University of Technology, Wiejska 45E Street, 15-351 Bialystok, Poland; 2 Department of Energy Systems Engineering, Faculty of Engineering, Izmir Institute of Technology, Urla, Izmir 35430, Turkey; gurayyildiz@iyte.edu.tr

**Keywords:** waste management, sustainability development, apple pomace, active compounds, extraction, renewable energy, anticorrosion agents, biopolymers

## Abstract

In many countries, apple pomace (AP) is one of the most produced types of agri-food waste (globally, it is produced at a rate of ~4 million tons/year). If not managed properly, such bio-organic waste can cause serious pollution of the natural environment and public health hazards, mainly due to the risk of microbial contamination. This review shows that AP can be successfully reused in different industrial sectors—for example, as a source of energy and bio-materials—according to the idea of sustainable development. The recovered active compounds from AP can be applied as preservatives, antioxidants, anti-corrosion agents, wood protectors or biopolymers. Raw or processed forms of AP can also be considered as feedstocks for various bioenergy applications such as the production of intermediate bioenergy carriers (e.g., biogas and pyrolysis oil), and materials (e.g., biochar and activated carbon). In the future, AP and its active ingredients can be of great use due to their non-toxicity, biodegradability and biocompatibility. Given the increasing mass of produced AP, the commercial applications of AP could have a huge economic impact in the future.

## 1. Introduction

Effective waste management is one of the greatest environmental challenges the world is facing today. Technological advancement, economic development, urbanization, population growth and consumer habits have significantly contributed to a rapid increase in waste generation. Moreover, there are no signs of deceleration of this trend in the near future [1,2]. The 2018 edition of *What a Waste 2.0: A Global Snapshot of Solid Waste Management to 2050* estimated that the global municipal solid waste production will be 2.01 billion tons per year, and is projected to grow to 3.4 billion tons per year by 2050. The largest amount of waste (approximately 44% of the total amount of waste generated in the world) is food and green waste [3]. Among them, large groups are by-products of the fruit processing industries, such as the apple juice industry, the disposal of the major by-products of which (i.e., apple pomace—AP) can pose serious environmental problems or even public health hazards if incinerated and/or dumped [4]. The global production of AP is estimated at an average of 4 million/year and is expected to have an increase in the future. Unfortunately, the recovery rate of AP is quite low and insufficient. The most commonly applied disposal method for AP is to discard it directly to the soil in a landfill. It may cause serious soil and water pollution because AP is rich in water (>70%), sugars and organic acids, which are susceptible to fast microbial fermentation. The growth in microbial flora may decrease available nitrogen in the soil and affect the C/N ratio. Moreover, some authors mention the potential toxicity of AP because apple seeds contain a cyanogenic glycoside, amygdalin. However, it is unlikely to cause acute cyanide poisoning in humans because this would require the consumption of 800 g of AP [5].

**Figure 1:**
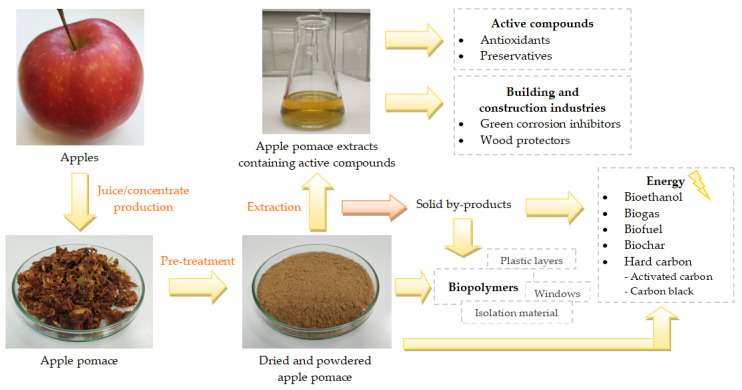
Application of apple pomace in production of green, non-toxic and biodegradable products with applications in construction and building.

Therefore, safe and efficient treatment and utilization of AP is required to reduce the possible environmental and health problems. Taking into account the huge amount of produced AP, the commercial applications of AP can create, in the future, great economic impacts. So far, different extraction methods that recover active substances from the AP have been applied. These active substances can later be applied as preservatives, antioxidants or anticorrosion agents used in, e.g., construction, civil engineering, environmental engineering and many other sectors (Figure 1). One of the basic techniques used to isolate the active compounds from materials of plant origin is classical extraction. In recent years, considerable attention has been given to the development of extraction methods that will be both efficient and environmentally friendly, e.g., limiting the amounts of solvent used or favouring energy efficiency [6]. Raw AP or the solid extraction by-products can also be utilised as a feedstock for the production of various types of intermediate bioenergy carriers in liquid, solid or gaseous forms, i.e., bioethanol, biodiesel, biomethane [7], biogas [8], biochar [9] or raw material for batteries [10]. Replacing traditional fossil fuels (FF) with AP-originated biofuels may reduce some undesirable aspects relegated to the production and use of FF, including emissions of greenhouse gases (GHG) (e.g., carbon dioxide (CO_2_) and nitrous oxide (N_2_O)), which contribute to serious environmental and health problems, and exhaustible resource depletion [11]. AP can also be a potential source of substances for the production of non-toxic and environmentally friendly biopolymers. The literature showed that AP-derived biopolymers were used for the production of biodegradable films, packaging materials, cups, plates and 3D objects [12,13].

Currently, due to poor waste management and a lack of environmental awareness in many countries, a large amount of apple pomace is treated as waste with no economic value. Besides, there are some technical limitations affiliated with the effective utilization of apple waste, such as the requirement of immediate treatment after obtaining it (e.g., by drying); this is important to prevent the excessive growth of microorganisms (microbiological contamination), and hence, the loss of overall economic value [14]. To foster sustainability, AP should be treated as a valuable raw material that can be reused or processed. In our work, the sustainable management of AP is based on the recovery and utilization of apple waste, which creates a possibility to reuse it and put it back into the supply chain. Green extraction techniques allow the obtaining of AP extracts that are rich in active compounds in an eco-friendly manner. The solid residues generated during extraction can be stabilised and further transformed into, e.g., alternative energy sources or biopolymers with zero waste. Therefore, sustainable AP management gives the opportunities for reducing environmental pollution and increasing integration into a circular economy [15]. This review presents the scale of AP production, and possible means of its utilization as a source of active compounds and biopolymers, a feedstock for the production of biofuels, and as a source of raw materials that can be utilised in different industrial sectors including the construction and building industry sector, the energy sector, and food or material industries.

### Apple and Apple Pomace Production in Poland and the World

**Figure 2:**
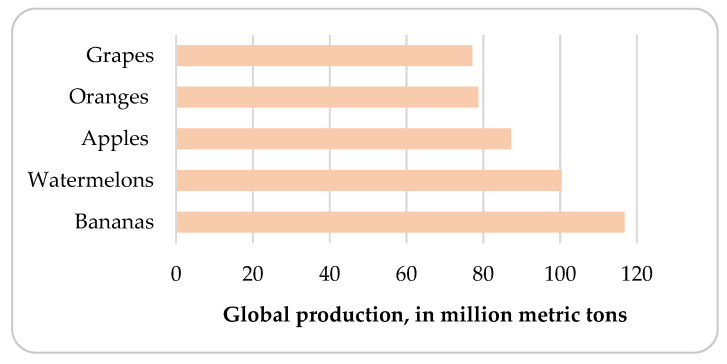
Global-scale production of the most popular fruits in 2019 [16].

According to the statistics, apples took third place (after bananas and watermelons), in 2019, in the terms of the popularity of fresh fruits in the world [16]. In that specific year, about 87.24 million metric tons of apples were produced worldwide (Figure 2). Poland is one of the largest producers of apples in the world (next to China, the United States and Turkey). From 2010 to 2018, the annual production of apples in Poland ranged from 1.877 to 3.9 million tons [16,17]. It is estimated that about 50% of all apples produced in Poland are processed for the production of apple juice concentrate [18]. Literature data show that 25–30 wt.% of the fresh apple used in the production of juice is a fruit by-product (i.e., apple pomace), which is considered a post-industrial organic waste [19]. Based on these facts, it can be calculated that about 0.5 million tons of AP were generated in Poland in 2018. For comparison, a neighbouring country, Germany, produces half as much AP (0.25 million tons/year) [20]. The literature shows that the largest apple producer, China, generates more than one million tons of apple pomace annually [21]. Countries such as New Zealand, Spain and Brazil are characterised by small amounts of generated apple juice by-products (from about 20,000 to 13,750 tons per year) [20]. Global apple production has reached over 87 million tons/year, resulting in 3.915–4.698 million AP (in 2019). Taking into account the fact that the production of 1 litre of apple juice requires about 1.6 kg of apples, 0.40–0.48 kg of AP is produced depending on the apple variety and processing type [22].

A report published by the Food and Agriculture Organization of the United Nations (FAO) indicates that approximately 33.3% (1.6 billion tons) of the food produced worldwide for human consumption is wasted each year [23]. High standards of product quality are required to attract consumers, and hence, the exclusion of the foods lower product quality is one of the major reasons. This problem particularly affects developed countries, including China, Japan, EU countries, Canada and the USA [24]. Such large losses pose a serious threat to the status of food security, the natural environment and the economy.

Fruits, including apples, that deviate from the imposed standards, for example in terms of size and visual elements (colour intensity, discolouration, skin elasticity, defects: bruises, rots) are often discarded by producers, consumers and retailers [25]. It is estimated that approximately 3.7 trillion apples end up in landfills each year [26]. Furthermore, such a waste of food and improper AP waste management causes huge losses of water, land, fertilizer, energy, labour and capital, which are economically unprofitable [24,27]. The amount of generated AP will increase each year due to the growing production of apples in orchards and the increased demand for processed products (juices, concentrates, jams and purees). To avoid the abovementioned problems, it is necessary to introduce economically viable pathways for waste apples and AP, so that they could be further processed into valuable products. Indeed, the cost-effectiveness of such pathways depends on the amount of waste generated/collected, the need for additional storage space and appropriate equipment (wet apple pomace require immediate processing due to high humidity) and related costs for transportation [28].

In this paper, we suggest a number of strategies to reuse the waste originating from apples. Indeed, fresh apples can also be treated as waste and used as renewable feedstocks in many industries, but this review focuses only on the use of AP produced by apple processing processes. In our opinion, AP should be reused according to the principles of the circular economy.

## 2. The Recovery from Apple Pomace Dried and Powder

### 2.1. The Pretreatment of Apple Pomace

#### 2.1.1. Biofuels

Due to serious concerns affiliated with climate change, the shift from fossil-based energy production options to various renewable-based alternatives has been a trending topic in the world. The most common renewable energy sources are wind, solar, hydropower, tidal, geothermal and biomass [29]. The latter involves organic materials such as agricultural residues, forestry residues, agri-food industry by-products (e.g., apple pomace), animal wastes, etc. Through various mechanical, biochemical or thermochemical processes, biomass can be transformed into fuels and/or fuel intermediates in solid (e.g., biochar), liquid (e.g., bioethanol, pyrolysis oil, etc.) and gaseous (e.g., biogas/biomethane, etc.) forms. Up to 80% of the organic matter in the pomace can be transformed into biofuel with an energy value of 10–30 W·m^−3^ [30]. Generally, production residues from the food industry are characterised by a low concentration of heavy metals and are good raw materials for biofuel production [31]. AP is rich in fermentable sugars (for example, it has contents of 19.2% fructose and 1.0% sucrose [32]) and is characterised by a low concentration of heavy metals; therefore it could be applied alone as a raw material for biofuel production. However, in practice, installations that use a mixture of several substrates are the most often used. Diversification of substrates favours the obtaining of better biofuel parameters and increases the safety of raw material supplies. Co-fermentation increases the efficiency of the process while reducing the costs incurred by the biogas plant for the purchase of raw material. Batches for energy production should be selected on account of, e.g., the maximization of energy yields, the stability of the fermentation process and the possibility of using the post-fermentation mass. In the study of Olech et al., the results of the analysis of the fermentation medium made of corn silage and apple pomace (in a proportion of 50/50%) showed that the highest methane generation was 61% [8]. The sample efficiency achieved a value of 4460 Nml. It was also demonstrated that olive and apple pomace are good co-substrate in the fermentation of cow slime (excrement) [33].

##### Bioethanol

Bioethanol (ethyl alcohol) is used as an eco-friendly fuel, most often as an additive to gasoline. It is obtained through the anaerobic fermentation of carbohydrates [34]. This process requires a proper pretreatment of the biomass feedstock (e.g., fruits) allowing the release of simple sugars (glucose, xylose, galactose, etc.) contained in cellulose and hemicellulose. This way, a hydrolysate rich in hexose and pentose sugars can be obtained [7]. In the case of anaerobic fermentation of the apple wastes, it is essential to choose an adequate bacterial strain to deal with a wide variety of sugars that are initially present in lignocellulosic hydrolysates. In addition to the commonly used yeast, *Saccharomyces cerevisiae*, there are references in the literature concerning the use of alternative microorganisms such as *Zymomonas mobilis*, *Kluyveromyces marxianus*, *Kluyveromyces lactis* or *Lachancea thermotolerans* for lignocellulosic biomasses such as apple pomace [7,35]. For example, the potential of apple pomace as a feedstock for bioethanol production was demonstrated in a recent study by Molinuevo-Salces et al. [7]. In their research, scientists assessed the effectiveness of selected bacterial strains on the amount of bioethanol produced from the hydrolysate of dry apple pomace obtained after juice extraction. The results showed that the highest bioethanol concentrations were obtained by testing *Kluyveromyces* sp. and *Lachancea* sp. (between 49.9 and 51.5 g L^−1^). Total sugar consumption was in the range of 74.5 and 80.0, with bioethanol yields from 0.402 to 0.444 g g^−1^ [7]. In the work of Demiray et al. [36], the influence of a cheap additive—soluble soy protein (at different concentrations: 20, 40, 80, 160 mg/g cellulose)—on enzymatic hydrolysis of AP was investigated. The results showed that the addition of 80 mg/g cellulose soluble soy protein to AP medium hydrolysed with 60 FPU (Filter Paper Units; enzyme concentration) increased the sugar (by 24.8%) and bioethanol concentration (by 8.28% in the case of *Saccharomyces cerevisiae*, and by 20.9% for *Kluyveromyces marxianus*), which makes the bioethanol production from AP process more efficient and still economical [36]. Kut et al. [37], for the first time, conducted the enzymatic hydrolysis of the liquid fraction of AP for the production of bioethanol using a pentose fermenter yeast, namely *Pichia stipitis*. The results of their research indicate that with a properly optimised process (10% (*w*/*v*) AP loading), about 84.1% of the theoretical ethanol yield can be obtained [37].

##### Biogas

Another type of alternative biofuel in gaseous form, biogas, is produced via a sequence of low temperature (ca. between 30 and 60 °C) processes by which certain microorganisms break down degradable biomaterials in the absence of oxygen. The produced gas mixture consists predominantly of methane (CH_4_) and carbon dioxide (CO_2_) with volumes of about 40–75% and 15–60%, respectively [38]. The production of biogas is not completely free of GHG, but research is underway to reduce the concentration of CO_2_ in biogas [39]; this increases the energy content of the final gas mixture and would result in increased levels of biomethane. Moreover, photosynthetic plants can absorb the CO_2_ released via biogas combustion, which results in the emission of less total atmospheric carbon than the classical combustion of coal [38]. In the last decades, the extensive amounts of globally generated apple wastes have received interest in terms of the exploration of their potential as a co-substrate to valorise biofuel production. For example, Olech et al. [8] showed that the anaerobic digestion medium made of apple pomace and corn silage (in the organic mass proportion of 50 to 50%) achieved a satisfactory level of methane yield (about 40%) on the third day of fermentation. The highest daily biogas yield was obtained on the ninth day of measurement and amounted to 4460 Nml [8]. In addition, after processing the fruit residues (with negligible heavy metal content) in biogas production, nutrient-rich organic fertiliser can be obtained [11]. In the study of Claes et al. [40], the influence of biochar and graphene (carbon-based conductive materials) and trace metals supplementation on biogas production from AP was investigated. The results of their study showed that this supplementation significantly improved the biogas production from the AP. At a COD (chemical oxygen demand) concentration of 6000 mg/mL, the addition of (a) trace metals, (b) biochar and (c) trace metals and biochar, increased the production of biogas by 7.2%, 13.3% and 22.7%, respectively, compared to the control (without supplementation). At a COD concentration of 12 mg/mL, the greatest changes in biogas production were observed for graphene supplementation (increase by 27.8% in comparison with the control). Moreover, in most cases, supplementation also improved the methane yield. In this study, the highest obtained CH_4_ yield (increased by 23.0% in comparison with control) was observed in a case of the reactor supplemented with biochar and trace metals (COD = 6000 mg/mL) (468.0 ± 3.6 mL CH_4_/g vs. and 286.0 ± 6.2 mL CH_4_/g COD) [40].

##### Biochar

Pyrolysis is a thermochemical process in which the organic material is decomposed into liquid (pyrolysis oil), solid (char) and gaseous (CO_2_, H_2_, CO, CH_4_) products. The process is carried out at moderate-to-high temperatures ranging from 300 °C to 650 °C and in the complete absence of oxygen. The type and particle size of biomass used, the heating rate and the residence times of, for instance, the feedstock and the generated primary pyrolysis vapours, are the major parameters that affect the performance and outcome of the pyrolysis process. The successful optimization of such process parameters determines the quantity, composition and quality of the products of the process [41,42]. There are several reports in the literature on AP pyrolysis (Table 1) [9,43,44,45]. In the work of Kosakowski et al. [43], the rapid pyrolysis of agricultural waste biomass (including AP) resulted in obtaining biochar characterised by higher combustion heat and calorific values than the biomass used [43]. Guerrero et al. [44] investigated the optimal conditions for the slow pyrolysis of AP for the production of gaseous products that can be used as a feedstock for the production of H_2_ [44]. Zhang et al. [45] used the biochar obtained in the AP pyrolysis process to create magnetic biochar that could effectively enrich the low concentration of Ag(I) ions in effluents [45]. Xu et al. [9] investigated the effect of the temperature and the type of biomass used on the production of biochar. The results showed that grape residues produced the highest biochar yield, while AP produced the least biochar [9]. According to Table 1, the biochar resulting from the pyrolysis/carbonization of AP had a net caloric value of between 25 and 31 MJ/kg. For comparison, a net value of good-quality milled coal, e.g., eco peat coal, is in the range of 24–26 MJ/kg [45].

#### 2.1.2. Sodium-Ion Batteries

Recently, there have been several reports published in the literature concerning the use of hard carbon (HC), obtained from fruit wastes and fruit peels, as an abundant and low-cost material for the production of sodium-ion batteries (SIBs) [10,46,47,48,49,50,51]. SIBs are cheap and environmentally friendly energy storage tools that are alternatives to the frequently used lithium-ion batteries (LIBs). Moreover, the vast abundance of sodium resources (the sixth most abundant element in the world) compared to the limited abundance of lithium and other elements commonly used in batteries, e.g., copper or nickel, also contribute to an increasing amount of research work being published [52,53]. In LIBs, graphite is used as an anode (negative electrode) material, while in SIBs, graphite is thermodynamically unstable with sodium ions [54]. A work published by Stevens and Dahn in the year 2000 [55] started the interest in hard carbon materials as potential anode materials for SIBs. In their work, the scientists demonstrated that this type of anode delivered a reversible capacity of 300 mAh·g^−1^, close to that obtained for graphite in LIBs (372 mAh·g^−1^) [55]. Hard carbon is usually prepared by the pyrolysis of organic precursors (most often from vegetable biomass, coal or petroleum) at temperatures between 1000 °C and 1500 °C, depending on the type of feedstock [56]. There are also some recent literature reports on the use of fruit waste as a source of hard carbon in SIBs. For example, in the study of Wu et al. [10], the electrochemical properties of apple waste-derived hard carbon electrodes were reported. Material for the electrodes (hard carbon) was obtained by a two-step dehydration process of wild apples followed by heat treatment (at 1100 °C) under an argon atmosphere. Then, the hard carbon electrodes (with a final composition of 80 wt.% HC) were prepared. The obtained electrodes demonstrated a very stable capacity of around 245 mAh·g^−1^ (at current rates of 0.1C) with full retention after 80 cycles, and good long-term cycling stability (1000 cycles at 5C). Moreover, the HC electrodes showed a promising rate capability with 112 mAh·g^−1^ at 5C [10]. Another study, conducted by Dou et al. [46], showed that pectin-free apple pomace waste-derived HC have a good overall performance during the galvanostatic long-term cycling at 0.1C (the capacity was around 285 mAh·g^−1^ after 230 cycles). Moreover, the specific capacities at 1.0–0.12 V (slope) and 0.12–0.02 V (plateau) (at 0.1C) during the fifth discharge were recorded. The obtained HC in the slope-like region showed a capacity of 110 mAh·g^−1^, while in the plateau region, a capacity of 175 mAh·g^−1^ was observed [46]. Interestingly, these results were quite different from those obtained for HC derived from apple pomace containing pectin in other works of the same author. HC from apple waste containing pectin delivered much lower capacity within the plateau as compared to HC from pectin-free apple waste (108 and 175 mAh·g^−1^, respectively). In the slope-like region, very similar capacities were recorded (112 and 110 mAh·g^−1^). This indicates the differences in the sodium storage mechanism of HCs [46,57].

#### 2.1.3. Biopolymers

Biopolymers are natural, biocompatible, highly biodegradable and environmentally friendly (“green”) alternatives to widespread synthetic plastics. Biopolymers can be obtained/extracted by the following means: *i.* from natural sources (e.g., agricultural waste); *ii.* via direct biosynthesis by microorganisms; and *iii.* through chemical synthesis [58]. AP is a promising raw material for the production of biopolymers due to its high sugar content. It is estimated that the dry mass of AP contains 7–44% cellulose, 14–17% starch, 15–20% lignin and 4–14% pectin, which can be used for the production of biopolymers [19].

There are several recently published literature reports regarding the production of sustainable biomaterials from AP [12,13,59,60,61,62,63,64]. In the study of Gustafsson et al. [13], AP was used for the production of 3D objects (fibreboards) and biofilms. Solution casting was used to form fibreboards, while film casting was used to produce biofilms. The obtained structures were tested for tensile strength (TS) and elongation at max (EAM). The results showed that the highest value of TS (5.79 MPa) and EAM (1.54%) was reached by the biopolymer made from AP with 30% (*w*/*w*) glycerol. For comparison, the biopolymer produced only from AP showed significantly lower values of TS = 3.71% and EAM = 1.56%. The biopolymer prepared from AP with 7% (*w*/*w*) glycerol had three-times-lower flexibility (EAM = 10.77%) and a four-times-higher TS value (TS = 16.49 MPa) than biofilm prepared from AP without glycerol using values of EAM = 37.39% and TS = 4.20 MPa [13]. In another work [59], AP-derived bioplastic was used in the production of cups. The mechanical properties of bioplastic were measured, and the results showed that the highest values of TS and EAM were also reached using a mixture of washed AP with 30% (*w*/*w*) glycerol content. However, other important parameters, such as water resistance, exposure to environmental factors (e.g., light), or biodegradability, were not investigated in this study. AP-derived biopolymer could be an environmentally friendly replacement for synthetic plastic tableware or additives for the production of structural or building elements (e.g., bricks) [59].

In the work of Liu et al., AP was characterised as a potential source of biopolymers—PHAs (poly-hydroxyalkanoates) [12]. PHAs are biosynthesised by a wide range of Gram-positive and Gram-negative bacteria (e.g., *Azotobacter*, *Clostridium*, *Alcaligenes latus* and *Cupriavidus necator*) and serve as an energy and carbon storage source [60]. Generally, the production of PHAs (2.4 and 5.5 US$/kg) generates much higher costs as opposed to conventional synthetic plastics (1.2 US$/kg) [61]. However, by changing the carbon source used in the production of PHAs to inexpensive agricultural waste (including AP), the production costs can be significantly reduced (up to 50%), which has a great impact on PHAs’ applicability in many industries [12,62].

In the work of Pereira [63], *Pseudomonas chlororaphis* sub-sp. *Aurantiaca* was used to produce medium-chain-length PHAs (mcl-PHAs) from apple waste. The obtained mcl-PHAs consisted of, i.e., 3-hydroxydecanoate (42.7 ± 0.1 mol%), 3-hydroxyoctanoate (17.9 ± 1.0 mol%), 3-hydroxybutyrate (14.5 ± 1.1 mol%) and 3-hydroxytetradecanoate (11.1 ± 0.6 mol%) with a yield of 49.25 ± 4.08%. The obtained mcl-PHAs biofilms showed attractive mechanical properties (TS = 5.21 ± 1.09 MPa, EAM = 400.05 ± 55.8%) [63]. Rebocho et al. [64], in their study, used apple waste as a feedstock for the production of mcl-PHAs using *Pseudomonas citronellolis*. The major components of the obtained biopolymer were 3-hydroxydecanoate (68% mol) and 3-hydroxyoctanoate (22% mol) with a total yield of 1.2 ± 0.05 (g/L). *P. citronellolis* mcl-PHA films showed high tensile strength (TS = 4.9 ± 0.68 MPa) and thermal stability [64]. The above research confirms the potential possibilities of using apple waste for the production of biopolymers that could be used as, e.g., packaging materials in many industries [63,64].

## 3. The Recovery from Apple Pomace Extraction for the Building and Construction Sectors

The considerable quantities of apple pomace produced in the world have been forcing researchers to develop novel and modern methods for their effective use. It is commonly known that apple peel (the main component of the pomace) contains a much higher content of active substances—phenolic antioxidants—than the pulp of the fruit. The relatively low price (compared to the price of raw apples) and widespread availability of AP make them a raw material with great potential [65,66]. However, due to their high water and sugar contents, AP are easily perishable (biologically unstable) and require immediate processing, such as dehydration (drying), as a pretreatment, which is associated with high energy consumption and, hence, additional OPEX. On the other hand, as an adverse side effect, drying can cause the degradation of temperature-sensitive valuable phenolic antioxidants [67]. The extraction of active compounds from AP can be an attractive method of their reuse. In addition, the solid waste generated during the process can be further used in accordance with the ideas of sustainability, e.g., as substrates for energy production.

### 3.1. Green Extraction Techniques

Green extraction techniques are methods for isolating active phenolic antioxidants from plant-based materials in an environmentally friendly manner. They rely on the utilization of alternative (green) solvents, eliminating the amount of synthetic and/or petroleum-based chemicals, and reducing energy costs and waste generation to obtain high-quality plant extracts [68]. Among green solvents, bioethanol is the most often used one due to its high biodegradability and low price [69]. Water, which is known to be the most natural solvent on the Earth, is effective only for the extraction of polar compounds [70]. Nowadays, various green extraction techniques including ultrasound-assisted, microwave-assisted, enzyme-assisted, pulsed electric field extraction, supercritical fluid extraction or pressurised liquid extraction have been explored [71]. Among them, supercritical fluid extraction (SFE), pressurised hot water extraction (PHWE), ultrasound-assisted extraction (UAE) or a combination of assisted extraction techniques are widely used. Recovered in a green way, phytochemicals from AP can be further used in many industries, including, e.g., construction and building, as anticorrosion agents, wood protectors, preservatives, antioxidants and biopolymers.

**Figure 3:**
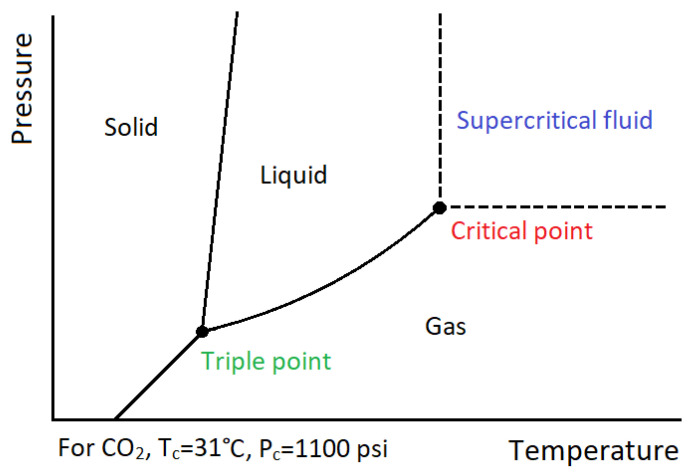
The phase diagram for CO_2_.

The SFE is a relatively new extraction method that is performed in the presence of supercritical fluids (most often liquid carbon dioxide—CO_2_). The process is carried out in specialised high-pressure equipment where CO_2_ is compressed under high pressure. The simultaneous increase in temperature and the pressure of the system leads CO_2_ to reach a supercritical state; in that phase, CO_2_ behaves similarly to both a liquid and a gas and mass transfer limitations that slow down the liquid transport are overcome [72]. After extraction, there is no need for additional purification of the extract or CO_2_ removal, because gas expands and evaporates at normal temperature and pressure (25 °C and 1 atm). Moreover, the SFE technique does not require air access, which protects the substances contained in the extracted material against oxidation. Another advantage is that the carbon dioxide (extraction solvent) used is non-toxic, odourless, colourless, non-flammable, cheap and reaches a supercritical state at relatively low temperatures (above 31 °C) (Figure 3). Due to such a relatively low temperature, it is possible to obtain plant extracts without losing their properties (degradation of active compounds), which often takes place at higher temperatures [73,74]. However, the SFE method has some disadvantages, with the main ones being the high cost of the aperture and the limited range of substances that can be extracted with CO_2_ as the sole solvent due to its non-polar nature [75]. The application of the SFE technique for the extraction of antioxidants from apple pomace was investigated in the work of Giovanna et al. [76]. In that study, fresh, freeze-dried and oven-dried apple pomace was treated with (a) subcritical CO_2_ and (b) subcritical CO_2_ with ethanol (5%) as a co-solvent, at pressures of 20 and 30 MPa and temperatures of 45 °C and 55 °C. For the comparison, a conventional extraction technology, i.e., Soxhlet extraction with ethanol and boiling water maceration, was also performed. The results of their research showed that the freeze-dried apple pomace extract obtained using the SFE method (at 55 °C, 30 MPa) with the use of ethanol (5%) as a co-solvent had the highest total phenolic antioxidant content (TPC) measured by the Folin–Ciocalteu method (8.87 ± 0.10 mg GAE (gallic acid equivalent)/g of extract) (Table 2). In addition, this extract was also found to have the highest antioxidant activity measured by DPPH^•^ assay (5.99 ± 0.11 mg TEA (Trolox equivalent antioxidant)/g of extract). For the extract obtained from SFE, carried out on freeze-dried apple pomace at the same conditions (55 °C, 30 MPa), but only with the subcritical CO_2_ as a solvent, the TPC was equal to 6.41 ± 0.19 mg GAE/g of extract. Much lower TPC was obtained for the freeze-dried extract obtained using the Soxhlet and boiling water maceration methods, with 4.13 ± 0.90 and 2.37 ± 0.01 mg GAE/g of extract obtained, respectively [76]. The optimal conditions for the SFE apple pomace extraction process were also investigated in the work of De la Peña Armada et al. [77]. The results of their research indicated that the optimal conditions for the SFE process could be established at a temperature of 46 °C and a pressure of 425 bar. Under these conditions, the obtained extracts were characterised by the highest concentration of triterpenic acids (betulinic acid, oleanolic acid, ursolic acid, uvaol, erythrodiol and lupeol) and the highest antioxidant activity tested by means of the ORAC (Oxygen Radical Absorbance Capacity) assay (609.17 ± 96.11 μmol TE (Trolox equivalent)/g extract). For comparison, the extract obtained using the Soxhlet method showed a lower antioxidant capacity (ORAC: 565.95 ± 60.66 μmol TE/g extract) [77].

PHWE is a similar technique to SFE, but in this case, water is used as a solvent. By increasing the temperature and pressure, water obtains similar properties to ethanol; this causes an increase in the solubility of many medium-polar compounds in water and ensures the extraction efficiency. The appropriate temperature of the extractant (water) should be above the atmospheric boiling point (100 °C, 0.1 MPa), but below its critical point (374 °C, 22.1 MPa) [78]. The main advantages of this method are its low cost and environmental friendliness; PHWE limits the use of organic solvents. Besides, the process water can be disposed of without causing any major environmental problems [79]. The applicability of the PHWE technique for the extraction of antioxidants from apple pomace was investigated in the work of Plaza et al. [79]. Using a response surface methodology (RSM), scientists optimised the PHWE parameters by maximising the yield of phenolic antioxidants from AP while minimising the possible formation of undesirable substances (e.g., melanoidins—the final Maillard reaction products). They reported that the highest amount of phenolic compounds (1.8 μmol/g dry AP) was obtained at a temperature of 170 °C and 3 min of extraction time [80].

**Figure 4:**
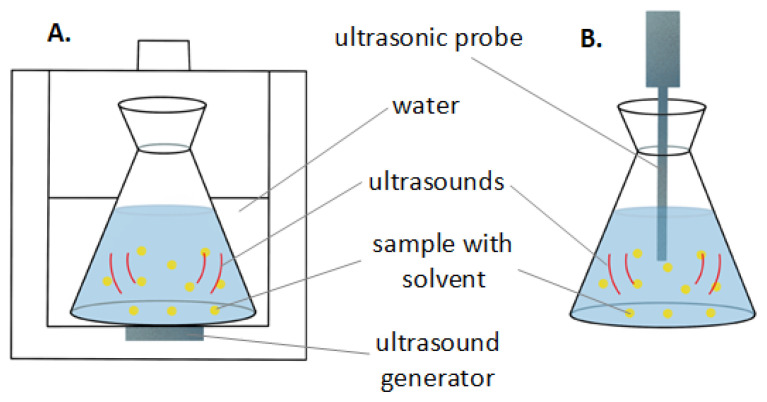
Ultrasonically assisted solvent extraction: (**A**) in an ultrasonic bath and (**B**) with a probe-generating ultrasound.

The UAE generates high-frequency pulses that increase the mass transfer of the extracted biocompounds to the used solvent. This is due to the presence of cavitation bubbles created by ultrasonic waves passing through the solvent. The rupture of cavitation bubbles on the analyte surface causes damage at the impact site and increases the rate of mass transfer of the extracted material to the solvent. UAE can be carried out using two types of device: ultrasonic (US) bath or probe-generating ultrasound (Figure 4). Both of them are equipped with one (US probe) or more (US bath) ultrasound generators called transducers. There is also a temperature control in the ultrasonic bath. Moreover, unlike extraction with a probe, several samples can be extracted simultaneously in an ultrasonic bath. Ultrasonic baths usually operate at frequencies from 37 to 45 kHz, while the ultrasound probes operate at a lower frequency of ca. 20 kHz. Lower frequencies lead to the formation of larger cavitation bubbles. The main disadvantage of this method is the possibility of partial degradation of the analyte compounds. In addition, after UAE, the obtained extract must be filtered to separate it from the extraction residues that sometimes require significant amounts of solvents and can lead to oxygen degradation of the extract. The UAE technique is often combined with other extraction methods, e.g., the Sono–Soxhlet approach involves the combination of UAE with Soxhlet extraction; other approaches include UAE being combined with microwave-assisted extraction, and the combination of UAE and SFE [81,82]. There are several reports in the literature from recent years on the use of the UAE technique to recover active substances from apple pomace [83,84,85,86]. For example, in the work of Pollini et al. [86], the effect of the solvent on the TPC of apple pomace extract was investigated. In their research, the extract obtained through UAE, using the mixture of ethanol and water (50:50, vv) as a solvent, had the highest TPC value (1062.9 ± 59.80 µg GAE/g of fresh AP) compared to other solvents used (ethanol:water, 70:30 and 30:70, vv) [86]. Malinowska et al. [84], compared the effect of the solvent used (water and ethanol) and the source of AP on the efficiency of the UAE process. The results showed that AP water extract (from conventional crops) had a two-times-lower TPC value than the AP ethanolic extract and the AP water extract (from ecological crops) [84]. The temperature of the UAE process, time of extraction and ultrasound power (e.g., power intensity) also plays an important role. Overly high temperatures (e.g., much higher than room temperature), power intensities (a wide range of ultrasonic frequencies of 20–100 Hz are applied in the literature) and expanded extraction times (time longer than 30 min) can lead to the deconstruction of valuable compounds [87,88]. The influence of the mentioned extraction conditions (temperature in the range of 10 °C to 40 °C, and ultrasound intensity in the range of 0.764 W/cm^2^ to 0.335 W/cm^2^) was studied in the work of Pingret et al. [88]. The results of their research indicate that the optimal conditions for the water-extraction of phenolic antioxidants from apple pomace using the UAE method are 40 °C, 40 min and 0.764 W/cm^2^ (Table 2) [89].

**Figure 5:**
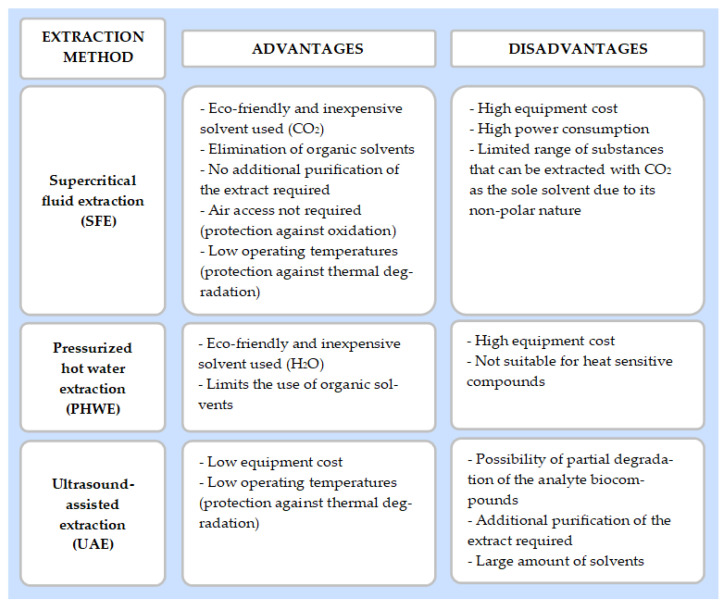
Comparison of the SFE, PHWE and UAE extraction techniques.

Regarding the evaluation of the above-mentioned extraction processes for the recovery of active compounds from apple waste, the efficiency and costs have to be evaluated. In the case of the SFE and PHWE techniques, the costs of extractions are relatively expensive, due to the high cost of specialistic equipment (Figure 5). However, the advantage of these techniques is that they use environmentally friendly solvents, such as CO_2_ and H_2_O. On the other hand, the cost of the ultrasonic bath or probe-generating ultrasound used in the UAE technique is relatively low, but this method requires larger amounts of solvents than in SFE and PHWE. However, taking into account the production costs of some synthetic compounds, obtaining compounds from the extracts may be a cheaper solution. Natural compounds are also more desirable than synthetic ones [90,91].

#### 3.1.1. Green Corrosion Inhibitors Active Compounds

Metals and their alloys have been widely used in the building and construction industries as base materials for various equipment (e.g., pipes and water tanks). However, factors such as moisture, salts, acidic and alkaline solutions, gases, etc. can lead to numerous damages to the material, known as the corrosion process [92]. The corrosion products (i.e., rust) significantly affect the construction elements and, hence, generate serious impacts on human safety and the overall economy of the construction process. Various methods are used to protect metal surfaces from corrosion. One of them is the use of substances that inhibit the corrosion process, i.e., corrosion inhibitors [93,94,95]. Recently, most of the commercially used corrosion inhibitors have been synthetic inorganic molecules containing, e.g., copper, zinc, arsenic, nickel or arsenic salts [96]. However, the use of most of them (e.g., toxic phosphate or chromates) raises concerns regarding the safety of living organisms and the natural environment (e.g., surface water) [94,97,98].

Nowadays, great emphasis in the construction industry is given to the use of natural, non-toxic, readily available and biodegradable products; therefore, new sources of substances that will be effective and inexpensive are considered [94]. Active molecules from AP extracts (phenolic compounds with antioxidant properties) are tested as potential corrosion inhibitors, due to their electron-donating properties and active sites [94]. Their anti-corrosion mechanism of action consists in the creation/adsorption of the protective film layer on the metal surface by blocking active sites on the metal surface (in response to the Langmuir adsorption isotherm). The inhibitory effectiveness is associated with the chemical composition of AP extracts and chemical structure of active phenolic compounds—the presence of heteroatoms, such as sulfur (S), oxygen (O), phosphorus (P) and nitrogen (N) in their polar functional groups (e.g., -OH, -COOH, -OCH_3_ -CN and -NO_2_). These heteroatoms favour the adsorption processes via an interaction between the metal surface and the π-electrons clouds in the conjugated system, or by the formation of the bonds with the non-bonding electron pairs of the heteroatoms [97,99,100,101]. Moreover, the corrosion inhibition efficiency (IE) of plant extracts is related to the electron density sites of the inhibitor molecules [96,97].

There are several reports in the literature on the use of AP extracts and individual active substances, that can be isolated from AP, as green anti-corrosion agents (Table 3) [97,100,102,103,104,105,106]. In the work of Vera et al. [100], the phenolic antioxidants occurring in the Fuji apple peel extract turned out to be highly effective (IE = 89.88% at an inhibitor/extract concentration of 1000 ppm) anticorrosive agents of carbon steel. The major components of the AP extract were 3,5,2′-trihydroxy-7,8,4′-trimethoxyflavone 5-glucosyl-(1->2)-galactoside (44.33%), 5-methoxy-6″,6″-dimethyl-3′,4′-methylenedioxypyrano (2″,3″,7,8) flavone (38.49%), quercetin-5-glucoside (3.27%) and quercetin-3-α-L-arabinopyranoside (3.15%). Other phenolic antioxidants, such as caffeic acid, chlorogenic acid, rutin, kaempherol and isoquercetin, were detected in lower concentrations [100]. In the study by Nazari et al. [97], an AP-based green inhibitor was found to exhibit high efficiency in reducing the carbon steel corrosion in 3.5% NaCl brine. 1-Linoleoyl-sn-glycero-3-phosphocholine (C_26_H_50_NO_7_P), containing N, P and O heteroatoms, was found to be a major constituent of AP extract (19.3 wt.%). The inhibition action mechanism of AP extract molecules was based on blocking the anode active sites on the steel surface and transforming Fe_3_O_4_ into a more corrosion-resistant Fe_2_O_3_. The highest IE (98%) was obtained on the seventh day of the measurement at the highest concentration of AP extract used (3%). In addition, the above-mentioned AP-derived inhibitor was synthesised without generating any waste [97]. In another study, pectin, which is abundant in AP, was used as an anti-corrosion coating for carbon steel. The protective effect (PE) increased with the increasing pectin concentration. For the lowest applied pectin concentration (100 ppm), PE = 83.62%, while for the highest (500 ppm), PE was equal 89.31% [102]. The influence of pectin on corrosion of metals in hydrochloric acid solution was also studied in the work of Fiori-Bimbi et al. [103]. In their work, the maximum value of pectin’s mild steel corrosion inhibition efficiency was equal to 94.2% (T = 318 K, inhibitor concertation = 2 g L^−1^) [102]. Pectin may also be a promising anti-corrosion agent for carbon steel in a neutral aqueous solution. Prabakaran et al. [104] developed an inhibitor composed of pectin (250 ppm), propyl phosphonic acid (50 ppm) and Zn(II) ions (20 ppm). The corrosion IE value for this mixture was 94%, indicating an excellent synergistic effect of components [104]. Procyanidin B2 and quercetin are major AP components. Procyanidin B2 was reported to be an effective corrosion inhibitor of carbon steel in 1 M HCl. The corrosion IE reached 94.21% at 30 °C (500 mg/L) after 24 h [105]. However, 800 ppm of quercetin was found to reduce 92% of mild steel corrosion in 1 M HCl after 1 h [106].

#### 3.1.2. Green Wood Protectors’ Active Compounds

Wood is a frequently used natural, renewable, relatively inexpensive and readily available building material used in the construction of structural beams, facilities, structures and wood objects (e.g., furniture and home decors). The use of wood in construction brings several benefits, e.g., wood is resistant to high temperatures, stretching (tensile strength) and electrical currents, it can absorb unwanted sounds (especially desirable in the construction of concert halls) and is highly machinable. Generally, wood can be divided into two types: hardwood (e.g., maple, oak, mahogany, beech and teak) and softwood (e.g., birch, pine and ash). Depending on the type, they differ in physical properties, such as density, strength, moisture content, etc. [107,108]. However, all of the types are exposed to factors such as weather conditions (moisture), fungi and insects, which contribute to its degradation [109].

Biological corrosion of wood causes significant changes in its structure, as well as in its chemical and psychical properties and can lead to complete material deconstruction (wood decay). To prevent wood and wood-based materials from these damages, various chemical wood preservatives are used. However, most of the traditional biocides used for wood protection are often highly toxic (Table 4) and can leach out from the preservative-treated wood, posing a serious risk to the environment, human and animal health [108,110]. For example, conventional synthetic wood preservative—CCA (Copper Chromium Arsenate)—contains arsenic and chromium (VI), which are easily leached from the wood surface and contaminate the surrounding soil. Arsenic is also known to be carcinogenic and, therefore, the use of CCA for wood conservation has been restricted since 2003 by the U.S. Environmental Protection Agency (EPA) [107,111]. To protect the environment and society, new alternative wood preservatives based on non-toxic and biodegradable natural substances should be developed. AP contains huge quantities of unused active substances, especially phenolic compounds, which are known to be potential antifungal and antibacterial agents [66,112].

Some types of wood (e.g., Alaska cedar, redwood) show natural resistance to insects, microorganisms and decay, due to the presence of extractives in hardwood [113,114,115]. Benzoic and cinnamic acids as well as their phenolic derivatives were found to be one of the extractive components responsible for the natural resistance of wood [116]. These compounds are plant secondary metabolites responsible for plant protection against biotic (insects, bacteria and fungi) and abiotic (drought, cold, heat and UV light) environmental stress [117,118]. Numerous studies have investigated the use of benzoic, cinnamic acids and their phenolic derivatives recovered from plant sources as potential natural and non-toxic wood protection agents (Table 5) [119,120,121,122,123,124]. The influence of benzoic, salicyli, syringic and vanillic acids on oil palm diseases caused by *Ganoderma boninense* was investigated in the study of Surendran et al. [119]. *G. boninense* is the major pathogen for basal stem rot (BSR) disease. Among all studied compounds, benzoic acid turned out to be the best *G. boninense* inhibitor. During all days of the measurement, benzoic acid at a concentration of 5 mM inhibited the growth of the tested pathogen. On the 120th day, the following weight loss was observed in the woodblocks treated successively with salicylic (≈34%), syringic (≈40%) and vanillic acids (≈75%) (C = 5 mM). For comparison, the mass loss of the untreated control woodblocks was 71.8% [119]. Sekine et al. [120], investigated the bioactivity of latifolin and its derivatives (Table 4) against termites and white- and brown-rot fungi. The results showed that latifolin exhibited significantly higher antifungal and anti-termite activity than the other tested compounds. For example, the value of inhibition rate of *T. versicolor* for latifolin was 79.1%, while for its derivatives, this was in the range of 13.2% to 21.8% [120]. In the studies of Little et al. [121], three flavonoids (quercetin, morin and catechin) and tannic acid were investigated as potential termite repellers. The results showed that wood treated with 3% tannic acid and 4% catechin caused high termite mortality—75% and 50%, respectively [121]. The anti-termite activity of flavonoids (apigenin, quercetin, biochanin A, genistein and taxifolin) was also reported in other works [122,123]. Efhamisisi et al. [124] impregnated 3-ply beech plywood with a mixture of 20% tannin solution and 1% boric acid (to enhance the crosslinking properties and prevent tannin loss). The results showed that such treatment significantly increased the resistance of panels against termites (*R. flavipes*) and fungal (*T. versicolor*) attack [124].

## 4. Conclusions

To improve food and environmental safety, it is important to properly manage agri-food waste so that it can be reintegrated into the existing bioeconomy [15]. The production of bio-waste, including apple pomace (AP), is expected to increase every year. Therefore, it is necessary to develop safe and effective methods for the processing and disposal of AP in accordance with the idea of sustainable development. The research results will allow for the acquisition of new knowledge as well as new bio-materials and technological solutions that could have a big economic impact in the future. Some of them are currently in use, e.g., green extraction techniques for the recovery of active substances from AP in an environmentally friendly manner, i.e., by lowering the energy consumption and reducing the amounts of harmful chemicals. Active compounds extracted from AP (including benzoic and cinnamic acid derivatives) can further replace the widespread synthetic chemicals and reduce the amounts of generated waste, e.g., they can be used as non-toxic, readily available and biodegradable anticorrosion agents or wood protectors in different industrial sectors. Through various processes (e.g., fermentation, anaerobic digestion and pyrolysis), AP can be transformed into fuels and/or fuel intermediates in solid (e.g., biochar-based hard carbon that can be used in Na-ion battery production), liquid (e.g., bioethanol, biodiesel, pyrolysis oil, etc.) and gaseous (e.g., biogas/biomethane, etc.) forms. Such transformations of apple waste into environmentally friendly energy and materials can not only reduce the consumption of conventional fossil feedstocks but also reduce the amount of GHGs emitted into the atmosphere. Finally, the AP can be considered as an environmentally safe biopolymer that can be applied as an innovative additive, e.g., in the production of structural or building elements or packaging materials in many other industries.

## Figures and Tables

**Table 1:** Apple pomace pyrolysis.

Product	Pyrolysis Type	Pyrolysis Parameters	Products Obtained	Net Caloric Values	Applications	Ref.
Apple pomace	Rapid	The temperature in the reactor was gradually increased to 850 °C over 30 min. Pyrolysis was continued for 60 min at a constant temperature. The experiments were carried out in triplicate with seven gas collection points (450, 515, 585, 650, 715, 785 and 850 °C).	Gas fraction: 47.5% Oil fraction: 14.1% Water: 12.6% Biochar: 25.9%	30.948 ± 168 kJ/kg (biochar) 19.775,2 ± 125,8 kJ/kg (biomass)	Biochar and gaseous products with sufficiently high combustion heat and net calorific values. Can be applied as additives in other fuels	[43]
Flavoured spirits production waste (FSW) (lime, grapefruit and lemon)			Gas fraction: 42.0% Oil fraction: 21.7% Water: 9.7% Biochar: 26.7%	26.598 ± 75 kJ/kg (biochar) 14.904,3 ± 553,8 kJ/kg (biomass)		
Beetroot pulp			Gas fraction: 32.9% Oil fraction: 24.6% Water: 12.3% Biochar: 30.2%	25.572 ± 139 kJ/kg (biochar) 15.169,2 ± 25,8 kJ/kg (biomass)		
Apple pomace	Slow	Temperature: 300–450 °C; heating rate: 5–20 °C/min; residence time: 60 min	CO, CO_2_, CH_4_	7.639,18 kJ/kg (AP)	Solid product yield is maximum in slow pyrolysis	[44]
Apple pomace	Not defined	Temperature: 600 °C; (pyrolysis followed by immersion ageing in Fe(II)/Fe(III) aqueous solution for obtaining AP-based magnetic biochar)	Magnetic AP biochar	Not defined	Magnetic AP biochar that can be used for enriching Ag(I) in effluents	[45]
Apple pomace	Not defined	Pyrolysis was carried out in a pilot bubbling fluidised bed pyrolyser operating under a range of temperature from 300 to 600 °C and vapour residence times ranging from 2 to 5 s.	Major gases: H_2_, CO, CO_2_, CH_4_; biochar; bio-oil	≈4–6 kJ/g (biomass)	A promising material for biochar production	[9]
Grape residues (GS—grape skins; GSS—grape skins and seeds)				≈0.1–4.1 kJ/g (biomass) ≈0.2–3.5 kJ/g (biomass)		

**Table 2:** The effects of different extraction techniques (conventional and unconventional) used for the recovery of biocompounds on the apple pomace extract composition.

Material for Research: Apple Variety, Sample Preparation	Extraction Method/Parameters	Analytical/Identification Method	Extract Composition/Identified Compounds	Antioxidant Activity	Ref.
Apple pomace separated from seeds and stems; a variety of apples not defined: (a) Fresh, only enzymatically stabilised; (b) Enzymatically stabilised, freeze-dried (−18 °C for 4h; 35 °C, 1.01 mbar for 20 h; 40 °C, 0.05 mbar for 6 h); (c) Enzymatically stabilised, oven-dried (50 °C for 4 days).	SFE; solvent: CO_2_; temp.: 45 and 55 °C; pressure: 20 and 30 MPa; extraction time: 120 min	Total phenolic content (Folin–Ciocalteu)	For 55 °C, 30 MPa: (a) 3.91 ± 0.27 mg GAE/g of extract; (b) 6.41 ± 0.19 mg GAE/g of extract; (c) 5.65 ± 0.14 mg GAE/g of extract	DPPH: (a) 1.93 ± 0.12 mg TEA/g of extract; (b) 3.24 ± 0.11 mg TEA/g of extract; (c) 2.72 ± 0.19 mg TEA/g of extract	[76]
	SFE; solvent: CO_2_ and ethanol (5%); temp.: 45 and 55 °C; pressure: 20 and 30 MPa; extraction time: 120 min		For 55 °C, 30 MPa: (a) 6.13 ± 0.16 mg GAE/g of extract; (b) 8.87 ± 0.17 mg GAE/g of extract; (c) 7.31 ± 0.18 mg GAE/g of extract	DPPH: (a) 2.67 ± 0.14 mg TEA/g of extract; (b) 5.99 ± 0.11 mg TEA/g of extract; (c) 4.73 ± 0.11 mg TEA/g of extract	[76]
	Soxhlet; solvent: ethanol; temp.: boiling temp. of ethanol; extraction time: 6 h		(a) 4.01 ± 0.06 mg GAE/g of extract; (b) 4.13 ± 0.90 mg GAE/g of extract; (c) 3.31 ± 0.12 mg GAE/g of extract	DPPH: (a) 1.96 ± 0.10 mg TEA/g of extract; (b) 2.05 ± 0.21 mg TEA/g of extract; (c) 1.38 ± 0.29 mg TEA/g of extract	[76]
	Boiling water maceration; solvent: water; temp.: 100 °C; extraction time: 37 min; 0.01 g/mL (solid-to-solvent ratio)		(a) 2.41 ± 0.01 mg GAE/g of extract; (b) 2.37 ± 0.01 mg GAE/g of extract; (c) 1.08 ± 0.11 mg GAE/g of extract	DPPH: (a) 1.17 ± 0.01 mg TEA/g of extract; (b) 1.14 ± 0.01 mg TEA/g of extract; (c) 0.92 ± 0.01 mg TEA/g of extract	[76]
Apple pomace constituted by seed, stalks, peel and a small proportion of pulp; “Golden Delicious” variety; apple pomace was freeze-dried at −45 °C and then milled to a fine powder	SFE; solvent: CO_2_; temp.: 37, 46 and 55 °C; pressure: 300, 425 and 550 bar; extraction time: 100 min	UHPLC (Ultrahigh-performance liquid chromatography)	Main compounds: betulinic acid, oleanolic acid, ursolic acid, uvaol, erythrodiol, lupeol	ORAC: 609.17 ± 96.11 μmol; TE/g extract (46 °C, 425 bar); HORAC: 104.83 ± 8.82 μmol; CAE/g extract (46 °C, 425 bar)	[76]
	Soxhlet; solvent: n-hexane; temp.: 70 °C; extraction time: 6 h		Main compounds: betulinic acid, oleanolic acid, ursolic acid, uvaol, erythrodiol, lupeol	ORAC: 565.95 ± 60.66 μmol; TE/g extract; HORAC: 193.20 ± 17.49 μmol; CAE/g extract	[76]
Apple pomace composed of seeds, cores, stems, skin and parenchyma; obtained from Kiviks Musteri in Kivik, Sweden	PHWE; solvent: n-hexane; temp.: 25, 50, 112, 175 and 200 °C; extraction time: 3, 5, 10, 15 and 17 min; extractions were performed in 11 mL extraction cells, containing 5 g of fresh sample	Total phenols concentration calculated by RSM	1.8 µmol/g of dry AP (170 °C, 3 min)	n.t.	[80]
Apple pomace; “Champion” variety; (a) conventional and (b) ecological crops; fresh apple pomace was stored at 4 °C for 24 h	UAE; solvent: water; temp.: 20 °C; extraction time: 30 min; solid/liquid ratio of 1:20 (g/mL); US bath (50 Hz, 300 W)	Total phenolic content (Folin–Ciocalteu)	(a) 14.33 ± 0.26 mg/l; (b) 31.28 ± 0.29 mg/l	n.t.	[84]
	UAE; solvent: ethanol; temp.: 20 °C; extraction time: 30 min; solid/liquid ratio of 1:20 (g/mL); US bath (50 Hz, 300 W)		(a) 28.46 ± 0.28 mg/l; (b) 44.34 ± 0.44 mg/l	n.t.	[84]
Apple pomace separated from seeds and petioles; “Red Delicious” variety; blended	UAE; solvent: ethanol and water in different ratios ((a) 50:50, (b) 70:30, and (c) 30:70, *v*/*v*); temp.: 60 °C; extraction time: 60 min; solid/liquid ratio of 1:10 (g/mL)	Total phenolic content (Folin–Ciocalteu)	(a) 1062.9 ± 59.80 µg GAE/g of fresh AP; (b) ≈ 900 µg GAE/g of fresh AP; (c) ≈ 800 µg GAE/g of fresh AP	n.t.	[86]
Apple pomace obtained from Val-de-Vire Bioactives (Conde-sur-Vire, France); kept in the dark	UAE; solvent: water; temp.: 40 °C; extraction time: 40 min; solid/liquid ratio 150 g/mL; US bath (25 kHz, 150 W)	Total phenolic content (Folin–Ciocalteu)	Predicted/calculated value: 555 mg of catechin equivalent/100 g of dry AP	n.t.	[89]

**Table 3:** Selected green corrosion inhibitors from AP.

Source	The Most Frequently Occurring Active Compounds	Metal and Electrolyte	Ref.
Fuji apple peel	3,5,2′-Trihydroxy-7,8,4′-trimethoxyflavone 5- glucosyl-(1->2)-galactoside, 5-Methoxy-6″,6″-dimethyl-3′,4′- methylenedioxypyrano(2″,3″,7,8)flavone	Carbon steel, 0.1 M NaCl	[100]
Apple pomace	1-Linoleoyl-sn-glycero-3-phosphocholine	Carbon steel, 3.5% NaCl	[97]
Pectin	-	Carbon steel, 1.0 M HCl	[102]
Pectin	-	Mild steel, 1.0 M HCl	[103]
Pectin	-	Carbon steel, H_2_O	[104]
Procyanidin B2	-	Carbon steel, 1.0 M HCl	[105]
Quercetin	-	Mild steel, 1.0 M HCl	[106]

**Table 4:** Examples of toxic substances used in wood preservatives [110].

Active Ingredient	Toxicity Class	Lethal Dose (LD_50_) * (mg/kg)	Main Use
Azaconazole	II. Moderately hazardous	308	Fungicide
Copper hydroxide	II. Moderately hazardous	1000	Fungicide
Copper oxychloride	II. Moderately hazardous	1440	Fungicide
Copper sulphate	II. Moderately hazardous	300	Fungicide
Chlorpyrifos	II. Moderately hazardous	135	Insecticide
Fipronil	II. Moderately hazardous	92	Insecticide
Thiamethoxam	II. Moderately hazardous	871	Insecticide
Disodium tetraborate (Borax)	III. Slightly hazardous	4500	Fungicide
Fenpropimorph	III. Slightly hazardous	3515	Fungicide
Tebuconazole	III. Slightly hazardous	1700	Fungicide
Dichlofluanid	U. Unlikely to present acute hazard in normal use	>5000	Fungicide
Fenoxycarb	U. Unlikely to present acute hazard in normal use	>10,000	Fungicide

* LD50—the amount of toxic substance (mg) per kg of body weight, which causes the death of 50% of a group of the tested animals.

**Table 5:** Phenolic compounds as natural wood preservatives.

Active Compounds	Wood Protected	Protection against	Results	Ref.
Benzoic acid Salicylic acid Syringic acid Vanillic acid	Oil palm (*Elaeis guineensis* Jacq.)	*Ganoderma boninense*	Controlled BSR disease.	[119]
Latifolin 2’-O-methyllatifolin Latifolin dimethyl ether Latifolin diacetate	Studies on paper discs	*Trametes versicolor* *Fomitopsis palustris* *Reticulitermes speratus* (Kolbe)	A significant activity of Latifolin against tested termites and fungi.	[120]
Quercetin Morin Catechin Tannic acid	*Pinus* sp.	*Reticulitermes flavipes*	A significant activity of catechin and tannic acid against tested termites.	[121]
Condensed tannin	European beach (*Fagus sylvatica* L.)	*Trametes versicolor* *Reticulitermes flavipes*	Increased resistance against tested termites and fungi.	[124]

## Data Availability

The data presented in this study are available on request from the corresponding author.
